# Understanding Longitudinal Muscle Injury Trends in Youth Football: Insights from U9 to U13 Players

**DOI:** 10.3390/sports13060163

**Published:** 2025-05-27

**Authors:** Jaksa Skomrlj, Toni Modric, Damir Sekulic, Mate Kuko, Luka Cikojević, Ante Bandalovic, Ante Turic, Boris Becir, Šime Veršić

**Affiliations:** 1 HNK Hajduk Split, 21000 Split, Croatia; skomrljj@gmail.com (J.S.); ante.bandalovic@hajduk.hr (A.B.); ante.turic@hajduk.hr (A.T.); boris.becir@hajduk.hr (B.B.); 2 Faculty of Kinesiology, University of Split, 21000 Split, Croatia; toni.modric@kifst.hr (T.M.); dado@kifst.hr (D.S.); mate.kuko@kifst.eu (M.K.); lukacikojevic33@gmail.com (L.C.); 3 High Performance Sport Center, Croatian Olympic Committee, 10000 Zagreb, Croatia; 4 Department of Orthopedics and Traumatology, Surgery Clinic, University Hospital Split, 21000 Split, Croatia

**Keywords:** injury incidence, academy, elite players, muscle injuries

## Abstract

This longitudinal study investigated the incidence and characteristics of injuries among U9, U11, and U13 male football players in an academy setting over a six-season period, from 2016/17 to 2021/22. A total of 374 injuries were analyzed, with a particular focus on muscle injuries, including Delayed Onset Muscle Soreness (DOMS), muscle ruptures, and contusions. The study revealed that the highest injury incidence occurred in the U13 group, with quadriceps injuries being most prevalent in both the U13 and U11 groups. The study found that muscle injuries accounted for a significant proportion of all injuries, particularly in the U13 group, where muscle injuries increased over time. Intrinsic factors such as physical development during puberty and extrinsic factors like training intensity and psychological pressures may contribute to the higher injury rates in older age groups. Additionally, seasonal fluctuations in injury rates were observed, with a notable decline during the COVID-19 lockdowns in 2019/20 and 2020/21, followed by an increase post-lockdown due to deconditioning. The study highlights the vulnerability of young athletes to muscle injuries, particularly during growth spurts, and calls for further research into training methods and injury prevention strategies to mitigate these risks.

## 1. Introduction

Youth football is experiencing a shift toward early specialization, with children engaging in structured training from a younger age [1,2]. While team sports like football have traditionally followed a later specialization model, modern training within structured academy environments emphasizes football-specific activities at an early stage, leading to higher training frequencies and longer cumulative training exposure [3,4]. Although accumulating sport-specific practice hours is beneficial for skill acquisition and cognitive development, excessive training loads have been linked to an increased risk of overuse injuries and early dropout [5,6].

The Long-Term Athlete Development (LTAD) model suggests that training volume, intensity, and complexity should progress gradually as athletes mature [7]. At the youngest levels (U9–U10), training primarily focuses on fundamental football techniques through play-based learning [8]. However, as players approach U12–U13, training becomes more structured, transitioning to senior-sized pitches (100–105 m × 64–68 m) with 11 vs. 11 matches of a standard 60 min duration. This shift, combined with physical maturation, leads to increased physiological demands and greater injury susceptibility [9].

A critical factor influencing injury risk in youth football is Peak Height Velocity (PHV), a phase of rapid somatic growth typically occurring earlier in female (between ages 11–13) than in male children (between ages 13–15) [10]. During this period, accelerated musculoskeletal development—characterized by sudden increases in bone length, muscle–tendon imbalances, and altered neuromuscular control—can predispose athletes to injuries [10]. Previous studies indicate that PHV coincides with a higher incidence of injury, particularly in the lower back, pelvis, knees, and Achilles tendon [11]. Additionally, increased training and match intensity during this phase further exacerbate injury risk [12]. This aligns with findings by Materne et al. [11], who reported significant variation in injury incidence across youth age groups.

Injury characteristics vary significantly across age groups. Young footballers (U9–U10) primarily suffer minor contusions and ankle sprains, with relatively low muscle injury prevalence [11]. However, from U10 onwards, there is a notable rise in growth-related injuries, likely due to biomechanical imbalances, such as altered neuromuscular control, muscle–tendon unit asymmetries, and poor coordination during growth spurts [13]. For U12 and U13 players, muscle injuries become more frequent, often resulting from increased training loads, weakened muscle structures, and growth-related changes in muscle–tendon dynamics [12].

Despite the growing body of research on youth football injuries, studies focusing specifically on muscle injury trends in pre-adolescent players remain scarce. Understanding how muscle injury incidence differs across age groups is crucial for optimizing training loads, reducing injury risk, and ensuring long-term player development. The aim of this study was to analyze muscle injury occurrence in U9–U13 players over six seasons, providing insights that can help coaches and medical staff refine training methodologies to enhance performance while minimizing injury risk. Unlike previous cross-sectional studies, the research team employed a longitudinal approach, allowing for the identification of seasonal patterns and developmental trends in muscle injuries across early age groups.

## 2. Materials and Methods

### 2.1. Study Design

This study investigated injury incidence in the U9, U11, and U13 groups, the three youngest age groups in the academic setting. Injury surveillance was carried out over a period lasting from the 2016/17 season until the 2021/22 season. The players who reported an injury were immediately examined by a medical specialist, and injury data were gathered systematically across the study period. Written informed consent was obtained from the parents or legal guardians of all participants, considering that all the players in the study were minors. This study was performed in accordance with the Declaration of Helsinki and was approved by the Ethics Committee of the Faculty of Kinesiology.

### 2.2. Participants

A total of 374 injuries among male U13, U11, and U9 players were analyzed in the study. Of the overall injuries, 151 injuries were to the muscles. The players in the study gradually progressed through the categories as they grew in age. In the studied academy, each generation had its own age group, but due to the needs of our study and the overall similarities among the groups, we merged/tied two generations into one age group (e.g., U8 and U9 are all considered the U9 group, U10 and U11 are all considered U11, etc.). The sample included players from all playing positions, regardless of the obvious positional differences and their respective playing demands. It is important to note that training and competition differ among the studied groups, with league formats varying slightly across age groups. For example, the U9 group averaged four 75 min training sessions with a 40 min match per week during 44 weeks of the season. The same applies to the U11 category, except for the training duration, which lasted for 90 min. As stated before, the most significant difference occurred in the U12 and U13 groups, with 60 min of gameplay on a full-size pitch. All players progressed chronologically through the groups, with position and playing time not being exclusion criteria. During the study, all the players were assigned a code to preserve the identity of the athletes.

### 2.3. Variables

A medical specialist was at the players’ disposal for a daily check-up. In this study, an injury is defined as any musculoskeletal complaint resulting from football training or match play that requires medical attention and/or leads to a restriction in participation, in line with the consensus definitions for injury surveillance in football [13]. Both traumatic and overuse injuries were included. Delayed Onset Muscle Soreness (DOMS) was classified as an injury only if it led to at least one missed training session or required medical treatment, following similar classification approaches used in other longitudinal injury surveillance studies. Although DOMS is a common adaptation to exercise, its inclusion reflects practical relevance in our academic setting, where significant soreness is documented and managed medically due to its impact on participation. Each injury was evaluated for severity (days absent), recurrence, contact nature, and activity type (training/match).

Immediately following the physical check-up, the injury data were available to all involved in the process (e.g., coaching staff and academy managers) through the club database. All injuries were recorded, with muscle injuries specifically classified as either (i) functional muscle injuries, represented by Delayed Onset Muscle Soreness (DOMS) and contusions, or (ii) structural injuries, i.e., ruptures (partial or complete tears of muscle fibers) [14]. Injury severity was calculated for all muscle injuries (i.e., DOMS, ruptures, and contusion injuries) in all age groups. Football exposure was calculated separately for all the observed seasons and age groups, and was reported as the number of training and match hours. Exposure time was adjusted for the 2019/2020 season, when a complete ban from all activities occurred during the COVID-19 lockdown (from March 2020 to July 2020). Muscle injury incidence was expressed as the number of muscle injuries sustained per 1000 h of training and match exposure. All muscle injuries were classified either as new or recurring injuries according to the body part, the contact nature of the injury, and the training or match occurrence. Regarding the injury site, muscle injuries were divided into groups representing the four most affected sites—hamstrings, quadriceps, adductors, psoas muscle—or a group comprising all other injured muscles.

### 2.4. Statistics

Descriptive data included arithmetic means and standard deviations. The muscle injury incidence for all age groups was presented as the number of injuries sustained per 1000 h of exposure, along with the incidence rate ratio that compared the absolute change in injuries across the seasons.

MedCalc Statistical Software (version 19.2.6), Microsoft Excel 2019 (Microsoft, Redmond, Washington, DC, USA) and a chi-square calculator (https://www.socscistatistics.com/tests/chisquare2/default2.aspx, accessed on 24 March 2025) were used for the analysis. The statistical significance was set at *p* < 0.05 for all calculations.

## 3. Results

A total of 151 muscle injuries (U13 = 108, U11 = 39, U9 = 4) were analyzed during the study. The results revealed the highest number of muscle injuries in the oldest category, U13 (N = 108), along with 39 muscle injuries in the U11 age category. The youngest age category, U9, suffered only 4 injuries to the muscular system.

Table 1 presents the descriptive data for all age groups. The majority of muscular injuries (64%) are characterized as DOMS injuries. Rupture injuries were the most serious, resulting in 25 days of absence on average for the U13 and U11 categories. No rupture was suffered in the U9 age group during the observed period. On average, players in the U11 category remained out of the training process for slightly longer than the U13 category (13.5 and 12.8 days average, respectively), with the U9 category losing one week of training per muscle injury. A higher number of training injuries was noticed in the U13 and U11 groups, in contrast to the U9 category, where 75% of muscle injuries occurred during the match. Approximately 21% of injuries happened after direct contact, either with the opponent or with the ball.

**Figure 1:**
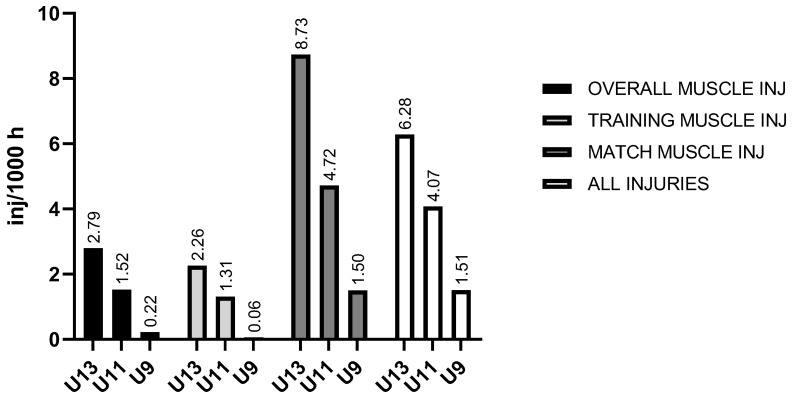
Muscle and total injury incidence per 1000 h across different age categories. Legend: inj/1000 h—injuries per 1000 h of exposure, U13—under 13, U11—under 11, U9—under 9.

The results suggest the highest muscle injury incidence occurred in the U13 group (=2.79/1000 h), with much higher match injury incidence than training injury incidence (=8.73/1000 h vs. =2.26/1000 h, respectively). Similarly, the match incidence is higher than the training incidence in the U11 and U9 groups as well (=4.72/1000 h vs. =1.31/1000 h, and =1.50/1000 h vs. =0.06/1000 h, respectively). Ultimately, the overall injury incidence follows the same pattern as above, with the numbers increasing with age (Figure 1).

**Figure 2:**
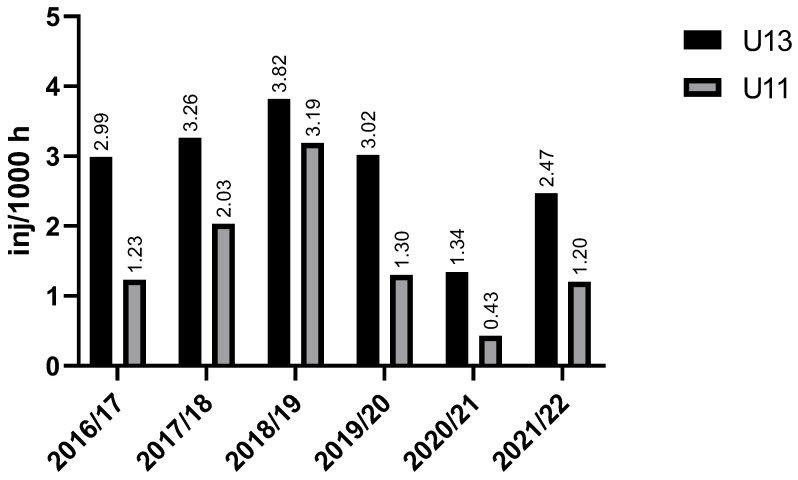
Injury incidence trends across seasons for U13 and U11 players. Legend: inj/1000 h—injuries per 1000 h of exposure, U13—under 13, U11—under 11.

There is no difference in muscle injury occurrence between the U13 and U11 groups across the seasons (chi-square (χ^2^) = 0.56; *p* = 0.99). A slight drop in injury incidence was found in in both groups during the 2019/20 and 2020/21 seasons (Figure 2).

**Figure 3:**
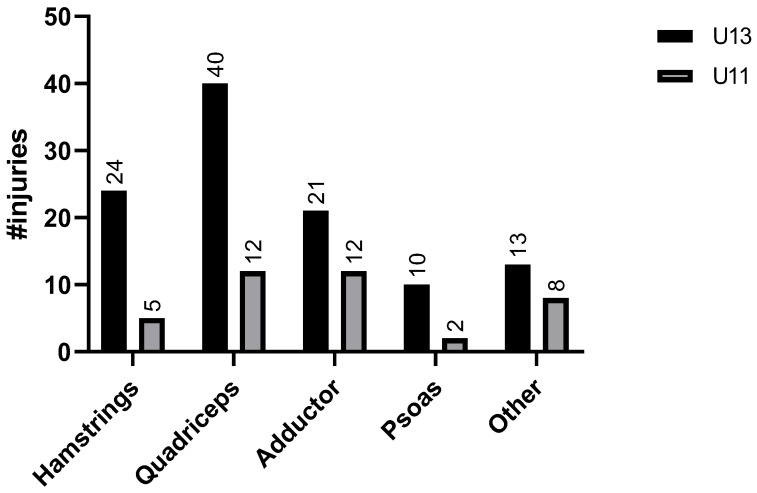
Distribution of muscle injuries by muscle group in U13 and U11 players. Legend: #injuries—number of injuries, U13—under 13, U11—under 11.

The U13 group suffered more muscle injuries compared to the U11 group. However, when comparing between the categories, no difference was found in the injury rates of different muscle groups. Regarding major muscle groups, the quadriceps were injured most frequently in both groups (Figure 3). Hamstring injuries were the second most common injury site in the U13 category, as opposed to adductors in the U11 group.

**Figure 4:**
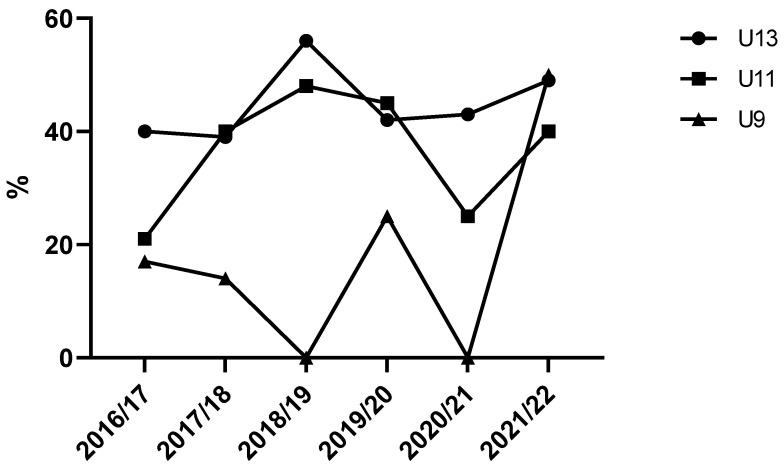
Percentage of injuries over time across age categories. Legend: %—percentage of muscle injuries among total injuries.

The proportion of muscle injuries in the total injury incidence follows a similar trend in the U13 and U11 age categories (Figure 4). Namely, there was a rise in the proportion of muscle injuries from the 2016/17 season until the 2018/19 season, followed by a drop during the next two seasons (i.e., seasons 2019/20 and 2020/21) and a final increase in the last investigated season. Not a single muscle injury was recorded in two out of the six observed seasons in the U9 category.

## 4. Discussion

This study aimed to investigate the overall injury and muscle injury characteristics among the three youngest academy age groups. According to the results, the most important finding is the highest injury incidence, which occurred in the oldest age category, U13. Further, the results suggest that the quadriceps were the most injured muscle in the U13 and U11 age groups. Finally, the proportion of muscle injuries was nearly the same in both age groups across the observed period.

### 4.1. Injury Incidence

The higher injury incidence observed in the U13 age group, particularly muscle injuries, may be partly explained by multiple intrinsic and extrinsic factors that have been highlighted in previous studies [12,13,15]. One of the primary contributors is the accelerated physical growth during puberty, which has previously been suggested to contribute to disproportionate development between bones, tendons, and muscles, increasing susceptibility to injury [13]. Additionally, the increased intensity of training and competition (i.e., transition to full pitch 11v11), coupled with greater psychological pressure, may further contribute to the higher risk of injury [15]. Moreover, as injuries are shown to increase with age, older athletes might have suffered a previous injury that predisposes them to a higher injury risk [16]. Although muscle injuries increased significantly in the U11 group (IRR 1.25) compared to U13 (IRR 1.15), the overall muscle injury incidence between these groups remained similar. This aligns with previous studies in English youth academies, where injury risk was found to double between U12 and U13 and increase significantly from U10 to U12 [17]. The relatively low injury rates in our study suggest that extreme deviations, such as injury spikes, are unlikely in this population.

Fluctuations in injury rates across seasons were influenced by several factors, including training intensity, competition load, playing surfaces, weather conditions, and player maturation stages [18,19]. Although not measured directly in this study, coaching changes have been linked to variations in muscular injury incidence, as different training methodologies, such as altered strength conditioning programs or biomechanical cueing, can impact injury rates [20].

The results indicated an increase in muscle injuries in the first half of the study period, both in the U13 and U11 age groups. Despite advancements in training technology, injury prevention strategies, and the increased involvement of medical professionals, injury rates in our academy increased slightly until the 2019/20 season [21]. This may be due to the reduction in muscle flexibility and strength during rapid skeletal elongation, a well-documented risk factor for muscle injuries [11].

The COVID-19 pandemic caused an unprecedented disruption in training, with lockdown measures leading to prolonged inactivity and detraining effects [22]. The lockdown measures were mandated in Croatia on two occasions during 2020/2021 as well, from December to January and from March to May. As a result, injury incidence dropped during the 2019/20 and 2020/21 seasons due to reduced training and match exposure. Similarly, studies investigating the pediatric population have shown a significant (i.e., up to 15%) reduction in injuries compared to the pre-pandemic season [23]. However, upon resumption of regular activities, a sharp increase in injuries was observed, likely due to deconditioning and loss of neuromuscular control [24]. This reinforces the importance of progressive return-to-play protocols following periods of inactivity to reduce injury risk [25]. Moreover, given that we are discussing children in their formative years, during which they should be consistently involved in vigorous physical activity, the evident risk posed to their development by multiple breaks in activity is clear.

### 4.2. Location and Type of Injuries

In youth football players, DOMS injuries are generally more common than ruptures, with muscle strains being more prevalent than ligament or tendon injuries [13]. Hence, emphasis should be placed upon a gradual progression of training load to prevent excessive muscle fatigue [7]. Among the U11 age group, injuries tend to result in longer absences, possibly due to these being the athletes’ first encounters with injuries and time away from training. This requires a more cautious and conservative approach to rehabilitation to ensure their proper recovery and return to the field. In addition, a recent study by Light and colleagues suggested an increase in total football exposure from 10.000 h to 22.000 h when moving from the U10 to the U11 age group [26]. Approximately 17% of injuries were recurrent, aligning with the 22–25% rates of reinjury reported in previous studies [27]. Further efforts should focus on addressing biomechanical deficits to reduce the risk of subsequent reinjury [21]. Training-related injuries were more common due to the significantly higher volume of training exposure, although the relative incidence is much higher during the matches [28]. Approximately 20% of injuries were contact-related, much lower than that reported in older age groups (U15–U19: 40–60%) [15]. This suggests that non-contact injuries play a more prominent role in younger players, highlighting the need for improved movement efficiency, neuromuscular control, and flexibility training. The injury locations across different age groups (U9, U11, and U13) showed no significant differences, with similar body regions and muscle groups being affected, as the same movement patterns (kicking, running, turning) are used across these age categories. The pattern of injuries remains largely constant, suggesting that movement-specific injuries are not significantly influenced by age [29]. In the U13 group, the quadriceps were most commonly injured, followed by the hamstrings, while U11 players experienced a similar number of quadriceps and adductor injuries. Consistent with the existing literature, the hamstrings, quadriceps, and hip region are particularly vulnerable to injury due to the high volume of multidirectional, high-speed movements in football [30]. Additionally, during the circa-PHV period, rapid growth and development place significant demands on the hip muscles, increasing their susceptibility to strain [31]. As young athletes are still developing strength and coordination, they may struggle with proper movement mechanics, which can put extra strain on these muscle groups [13]. Therefore, structured eccentric strength training should be introduced to circa-PHV athletes to enhance muscle resilience at key sites [6].

### 4.3. Proportion of Muscle Injuries

A similar trend in muscle injuries was observed across the two older age groups, with nearly identical injury incidences in three out of six seasons. The proportion of muscle injuries rose consistently (in the U11 and U13 age groups) until the 2019/20 season, when COVID-related disruptions impacted the data. A similar pattern is observed throughout the research period, with both the overall injury incidence and the proportion of muscle injuries increasing. In the context of analyzing the relative proportions in the overall injury dataset, this suggests that muscle injuries increased at a faster rate than total injuries, leading to a larger share of muscle injuries within the overall injury pool. On average, the proportion of muscle injuries was 45% for the U13 group and 37% for the U11 group, which aligns with previous findings in elite youth footballers, where muscle injuries accounted for 45% of the total injuries [32]. Although training technology has advanced and conditioning coaches have been added to support these age groups, muscle injuries have continued to rise. Therefore, it is crucial to further investigate the appearance and mechanisms of injuries, as well as the risk factors in this young and vulnerable population. A potential solution could lie in the use of objective measures such as GPS and RPE to track and monitor workload [11]. This could offer insights into the appropriateness of training loads and highlight periods of excessive strain that may predispose these players to injury. Interestingly, no muscle injuries were recorded in the U9 group during two out of the six seasons, highlighting the already low injury incidence in this age group [26].

### 4.4. Strengths and Limitations

One of the key limitations of this study is the inability to control for other potential influencing factors, such as maturity status or bio-motor abilities, which could significantly impact the incidence of muscle injuries. These factors, while not addressed in the current study, are crucial in understanding the complex relationship between physical development and injury risk. Additionally, the study’s focus on a single football academy limits its generalizability, as the results may not apply to other contexts or regions. Despite these limitations, the study’s longitudinal design, spanning six years and involving a large sample size in an elite youth academy, provides a clear insight into muscle injury occurrence in the U9 to U13 age groups. The emphasis on the youngest age groups is a major strength, as it identifies the most vulnerable regions of the body that are prone to injury during this critical developmental phase. By identifying these early risks, the study provides a foundation for proactive measures to safeguard young athletes’ long-term health and development.

## 5. Conclusions

This study highlights the higher injury incidence in the U13 age group, likely due to increased training intensity, rapid physical development, and higher psychological pressure. Muscle injuries, particularly in the quadriceps, were most prevalent in the U13 and U11 groups, with the proportion of muscle injuries rising steadily over the study period. Although training methods have advanced and conditioning coaches have been introduced, muscle injury rates remain high, particularly among the U13 and U11 groups, indicating that the rapid maturation process during these years increases susceptibility to injury. The findings emphasize the importance of age-specific injury prevention strategies, particularly during periods of significant physical development. For younger athletes (U9–U11), emphasis should be placed on developing fundamental movement skills, balance, and coordination through neuromuscular training. As athletes progress (U13–U15), injury prevention should include progressive strength training targeting key muscle groups, alongside refining movement mechanics such as landing and cutting techniques. In older youth athletes (U16+), sport-specific conditioning and individualized load management become essential to the prevention of overuse injuries. Overall, improving movement competency, running mechanics, and structured strength training can significantly reduce injury risk in young athletes. Future research should aim to control these confounding variables and explore other significant predictors of injury more thoroughly.

## Figures and Tables

**Table 1:** Descriptive data for muscle injuries across age categories.

		# Injuries	Days Out	Reinjury	Occurrence		Contact
					Training	Match	
U13	Rupture	27	24.5	14	18	9	5
	DOMS	68	8.4	3	54	14	5
	Contusion	13	5.4	4	8	5	13
	Total	108	12.8	21	80	28	23
U11	Rupture	12	25.5	2	8	4	4
	DOMS	26	8.9	4	20	6	2
	Contusion	2	6	0	2	0	2
	Total	39	13.5	6	30	10	8
U9	Rupture	/	/	/	/	/	/
	DOMS	4	7.3	0	1	3	0
	Contusion	/	/	/	/	/	/
	Total	4	7.3	0	1	3	0

Legend: # INJURIES—number of injuries.

## Data Availability

The data are available upon reasonable request.
